# Spontaneous Bacterial Peritonitis: A Rare Incidence by Achromobacter xylosidans

**DOI:** 10.7759/cureus.67855

**Published:** 2024-08-26

**Authors:** Paul Q Vu, Mrudula Thiriveedi, Siddharth Patel, Kalashree Gopal

**Affiliations:** 1 Internal Medicine, Alabama College of Osteopathic Medicine, Dothan, USA; 2 Internal Medicine, Decatur Morgan Hospital, Decatur, USA

**Keywords:** secondary bacterial peritonitis, s: nosocomial infections, multidrug-resistance, achromobacter xylosidans, gram negative bacteremia, cirrhosis, ascites, spontaneous bacterial peritonitis (sbp)

## Abstract

Liver cirrhosis results from progressive hepatic fibrosis and is generally considered irreversible. One of the many consequences of cirrhosis is spontaneous bacterial peritonitis. This typically presents in patients with decompensated cirrhosis due to bacterial translocation, most commonly from the intestinal bacterial flora seeding into the ascitic fluid. We present a rare case of spontaneous bacterial peritonitis caused by *Achromobacter xylosidans.* This bacterium is mostly associated with nosocomial infections, and due to its multidrug-resistant nature, treatment options are often limited. This case highlights a rare cause of spontaneous bacterial peritonitis to consider in the setting of recent hospitalization, and the importance of recognizing spontaneous bacterial peritonitis versus secondary bacterial peritonitis.

## Introduction

Cirrhosis is an irreversible, late-stage disease progression of hepatic fibrosis. Hepatitis C, alcohol-associated liver disease, and nonalcohol-associated fatty liver disease together are the leading cirrhosis-associated causes of morbidity and mortality in the United States [1]. Cirrhosis is an indolent disease and patients are often asymptomatic until they develop complications; these can include ascites, variceal hemorrhage, hepatic encephalopathy, hepatocellular carcinoma, hepatorenal syndrome, hepatopulmonary syndrome, and spontaneous bacterial peritonitis (SBP). The focus of this article is to discuss SBP, which typically occurs when ascitic fluid becomes infected. SBP is believed to occur from the translocation of bacteria from the intestines which are usually enteric gram-negative bacteria, such as *Escherichia coli* and *Klebsiella *[2]. We present a rare case of SBP caused by *Achromobacter xylosidans*. This bacterium is an aerobic, non-glucose fermenting, gram-negative bacillus that is a potential cause of catheter-associated bacteremia [3]. Most cases are described in patients with immunosuppression due to hematological malignancies [4]. Interestingly, in our case, the patient did not have any underlying medical conditions aside from liver cirrhosis. *Achromobacter xylosidans* is also intrinsically resistant to carbapenems resulting in devastating consequences due to multidrug resistance [4].

This article was previously presented as a poster at the University of Alabama at Birmingham, Huntsville Regional Campus Research Day in April 2020.

## Case presentation

A 47-year-old African American female with a history of significant alcohol use and recently diagnosed decompensated cirrhosis presented to the emergency room with altered mental status (AMS). She was hospitalized two weeks prior to this admission with ascites and underwent paracentesis which did not reveal evidence of infection. The patient was hypotensive, tachycardic, and tachypneic upon arrival. She was also noted to be lethargic with a distended abdomen on physical examination. Laboratory analysis was significant for leukocytosis, thrombocytopenia, hypoglycemia, acute kidney injury, lactic acidosis, low albumin, and elevated total bilirubin, international normalized ratio (INR), and ammonia levels (Table 1). Ascitic fluid analysis revealed cloudy fluid with a total nucleated count of 49,300 cells/mm^3^ and neutrophil predominance of 90%, total protein of 1.8 g/dL, glucose of less than two, and lactate dehydrogenase (LDH) of 474 U/L (Table 2). Computed tomography (CT) scan of the abdomen and pelvis was obtained to rule out secondary causes of peritonitis given her ascitic fluid findings. There was no evidence of perforation or abscess found. The patient was admitted to the intensive care unit (ICU) for sepsis from bacterial peritonitis and was initiated on intravenous fluids, intravenous cefepime, and albumin. A Foley catheter was also placed for intake and output monitoring.

**Table 1:** Laboratory Analysis on admission and two weeks prior

Test:	Results:		Reference range:
	On Admission	Two Weeks Prior	
Lactate	17 mmol/L	2.1 mmol/L	0.7-2.1 mmol/L
White Blood Cell (WBC) Count	18,530 cells/μL	11,540 cells/μL	4000-11,000 cells/μL
Platelets	45,000 cells/μL	-	130,000-400,00 cells/μL
Sodium	134 mmol/L	137 mmol/L	136-145 mmol/L
Blood Glucose	40 mg/dL	182 mg/dL	70-99 mg/dL (fasting)
Creatinine	1.8 mg/dL	0.5 mg/dL	0.50-1.10 mg/dL (female)
Total Bilirubin	13.1 mg/dL	5.1 mg/dL	0.3–1.0 mg/dL
Albumin	2.8 g/dL	2.8 g/dL	3.5–5.5 g/dL
International Normalized Ratio (INR)	3.9	2.6	0.9-1.1
Ammonia	119 μg/dL	-	11–32 μg/dL

**Table 2:** Ascitic Fluid Analysis via Paracentesis on admission and two weeks prior

Test:	Results:		Reference range:
	On Admission	Two Weeks Prior	
Total Nucleated Count (TNC)	49,300 cells/mm^3^ (90% neutrophils)	60 cells/mm^3^	<250 cells/mm^3^
Total Protein	1.8 g/dL	1.5 g/dL	>1g/dL: consistent with secondary bacterial peritonitis <1 g/dL: consistent with spontaneous bacterial peritonitis
Glucose	< 2 mg/dL	119 mg/dL	>50 mg/dL
Lactate Dehydrogenase (LDH)	474 U/L	47 U/L	<225 U/L
Fluid Albumin	0.7 g/dL	0.7 g/dL	-
Serum Ascitic Fluid Albumin Gradient (SAAG)	2.1 g/dL	2.1 g/dL	<1.1 g/dL

In the ICU, she had refractory lactic acidosis and hypoglycemia, likely due to liver failure, and was treated with sodium bicarbonate and dextrose drips. The patient also received lactulose enemas for hepatic encephalopathy. The Model for end-stage liver-sodium (MELD-Na) score worsened to 37 from 23 two weeks ago. An ascitic fluid culture and two sets of blood cultures grew *Achromobacter xylosidans* which showed intermediate sensitivity to cefepime, and sensitivity to piperacillin/tazobactam and levofloxacin. The patient was not deemed to be a candidate for liver transplantation given her last alcoholic drink was three months ago in addition to the presence of bacteremia. Her clinical status worsened requiring vasopressor support and eventually, the family decided to transition her to comfort measures only.

## Discussion

Cirrhosis occurs when the hepatic architecture is distorted with formation of regenerative nodules from progressive hepatic fibrosis. It was the eighth leading cause of death in the United States in 2010 and 2019, it was the leading cause of death worldwide (2.4% of global deaths) [5-6]. Cirrhosis can develop from a single insult or be a result of multifactorial issues. Common etiologies of cirrhosis include alcohol use (prevalence of 60-70%), chronic hepatitis C (10%), and nonalcoholic fatty liver disease (10%) [1]. Patients with compensated cirrhosis may be asymptomatic or report nonspecific symptoms such as anorexia, weight loss, weakness, and fatigue, while those with decompensated cirrhosis may present with jaundice, pruritus, upper gastrointestinal bleeding, abdominal distension from ascites, or confusion.

SBP is one of the many devastating complications that can lead to hepatic decompensation in cirrhotic patients. SBP accounts for 10 to 40% of all hospitalized cirrhotic patients with ascites and has a one-year mortality after the first episode of 30% to 90% [7-8]. SBP is defined as an ascitic fluid infection without an evident intra-abdominal surgically treatable source. It is diagnosed when the ascitic fluid absolute polymorphonuclear leukocyte (PMN) count is greater than 250 cells/mm^3^, the ascitic fluid cultures are positive, and the secondary causes of peritonitis are excluded [9]. The proposed pathogenesis of SBP starts with bacterial entry into the ascitic fluid, most commonly from organisms in the gut flora (eg, *Escherichia coli*, a prevalence of 43%) [10]. A common site of bacterial translocation is the mesenteric lymph nodes and ultimately, the bacteria will enter the bloodstream leading to bacteremia and eventually infection of the ascitic fluid resulting in SBP [11]. Patients with decompensated cirrhosis are more likely to develop infections due to impaired neutrophilic and macrophagic activity, as well as issues with complement activation [12]. Thus, the impaired host immune defenses allow intestinal bacterial overgrowth of pathological strains and increased intestinal permeability, which is thought to promote bacterial translocation and the development of SBP [13].

SBP should be suspected in cirrhotic patients with ascites who develop fever, abdominal pain/tenderness, and AMS. Our patient initially presented two weeks prior with AMS and was diagnosed with decompensated alcoholic cirrhosis. Ascitic fluid evaluation at that time was consistent with portal hypertension and did not show any evidence of infection. During the present admission, our patient’s vital signs were unstable, and she was lethargic with a distended abdomen. Laboratory analysis showed numerous derangements as described above with a leukocyte count of 18,530 cells/μL, INR of 3.9, and ammonia of 119 μg/dL. These results indicated an infectious etiology in the setting of acute hepatic failure. Ascitic fluid analysis revealed significantly elevated absolute PMN count, low glucose, and elevated LDH. The ascitic fluid culture and two sets of blood cultures grew *Achromobacter xylosidans* which is a rare cause of peritonitis and bacteremia. This bacterium is most commonly associated with malignancies (prevalence of 30%), cardiac disease (21%), and immunosuppression (27%) [14-15].

*Achromobacter xylosidans* usually causes nosocomial bacteremia (prevalence of 70%) associated with catheters (19%) [15]. Other reported sites of infection include meningitis, urinary tract infection, abscesses, osteomyelitis, corneal ulcers, prosthetic valve endocarditis, and pneumonia [4]. Our patient did not have any underlying malignancies or cardiac disease. Her only medical condition was cirrhosis which may have contributed to her immunocompromised state. Our patient was hospitalized two weeks prior and underwent paracentesis which may have introduced the bacteria into the bloodstream causing bacteremia, seeding in the ascitic fluid, and ultimately development of SBP. Her Foley catheter could have also been another potential source of infection. Of note, while in our case, *Achromobacter xylosidans* was sensitive to piperacillin/tazobactam, it can be difficult to treat due to developing multidrug resistance [16]. In patients with recent hospitalization, decompensation, and concern for multidrug-resistant pathogens, utilization of chronic liver failure-sequential organ failure assessment (CLIF-SOFA) score may help determine the best course of empiric antibiotic treatment [17].

Another important thing is to determine whether our patient had spontaneous bacterial peritonitis (SBP) or secondary bacterial peritonitis. This differentiation is crucial to decrease mortality in cirrhotic patients. For example, the mortality of SBP in patients receiving unnecessary exploratory laparotomy is approximately 80% while the mortality of secondary bacterial peritonitis with only antibiotics and no surgical intervention nears 100% [18-19]. Diagnosis of secondary bacterial peritonitis includes at least two of the following ascitic fluid findings: Total protein greater than 1 g/dL (10 g/L), Glucose less than 50 mg/dL (2.8 mmol/L), or LDH greater than the upper limit of normal for serum [19]. Our patient met all the criteria for secondary peritonitis however CT of the abdomen and pelvis did not show any evidence of perforation or abscess. Also, cultures of ascitic fluid for secondary bacterial peritonitis are typically polymicrobial while cultures of that for SBP are typically monomicrobial [20]. The cultures obtained from our patient were monomicrobial and only grew *Achromobacter xylosidans* which led to the diagnosis of SBP.

## Conclusions

Patients with cirrhosis can decompensate and develop devastating complications such as spontaneous bacterial peritonitis. Clinical suspicion of spontaneous bacterial peritonitis should arise in cirrhotic patients who present with fever, abdominal pain, and altered mental status. *Achromobacter xylosidans* is a rare cause of spontaneous bacterial peritonitis and is difficult to treat due to its multidrug resistance nature. It may be beneficial to assess a patient with cirrhosis utilizing the chronic liver failure-sequential organ failure assessment score to guide empiric antibiotic coverage, especially for patients in the setting of recent hospitalization and concern for multidrug-resistant pathogens. Furthermore, it is imperative to distinguish spontaneous bacterial peritonitis and secondary bacterial peritonitis to guide management and decrease mortality.
